# IGF-1-mediated FOXC1 overexpression induces stem-like properties through upregulating CBX7 and IGF-1R in esophageal squamous cell carcinoma

**DOI:** 10.1038/s41420-024-01864-0

**Published:** 2024-02-27

**Authors:** Hao Wu, Zhao-Xing Li, Kang Fang, Zi-Ying Zhao, Ming-Chuang Sun, An-Qi Feng, Zhu-Yun Leng, Ze-Hua Zhang, Yuan Chu, Li Zhang, Tao Chen, Mei-Dong Xu

**Affiliations:** 1 Endoscopy Center, Department of Gastroenterology, Shanghai East Hospital, School of Medicine, Tongji University, 200120 Shanghai, China; 2 Department of Pathology, Shanghai East Hospital, School of Medicine, Tongji 8 University, 200120 Shanghai, China

**Keywords:** Oesophageal cancer, Cancer stem cells

## Abstract

Substantial evidence attests to the pivotal role of cancer stem cells (CSC) in both tumorigenesis and drug resistance. A member of the forkhead box (FOX) family, FOXC1, assumes significance in embryonic development and organogenesis. Furthermore, FOXC1 functions as an overexpressed transcription factor in various tumors, fostering proliferation, enhancing migratory capabilities, and promoting drug resistance, while maintaining stem-cell-like properties. Despite these implications, scant attention has been devoted to its role in esophageal squamous cell carcinoma. Our investigation revealed a pronounced upregulation of FOXC1 expression in ESCC, correlating with a poor prognosis. The downregulation of FOXC1 demonstrated inhibitory effects on ESCC tumorigenesis, proliferation, and tolerance to chemotherapeutic agents, concurrently reducing the levels of stemness-related markers CD133 and CD44. Further studies validated that FOXC1 induces ESCC stemness by transactivating CBX7 and IGF-1R. Additionally, IGF-1 activated the PI3K/AKT/NF-κB and MEK/ERK/NF-κB pathways through its binding to IGF-1R, thereby augmenting FOXC1 expression. Conversely, suppressing FOXC1 impeded ESCC stemness induced by IGF-1. The presence of a positive feedback loop, denoted by IGF-1-FOXC1-IGF-1R, suggests the potential of FOXC1 as a prognostic biomarker for ESCC. Taken together, targeting the IGF-1-FOXC1-IGF-1R axis emerges as a promising approach for anti-CSC therapy in ESCC.

## Introduction

Esophageal cancer is a prominent contributor to global cancer-related mortality, with esophageal squamous cell carcinoma being its predominant histological subtype. Ranking as the 6th most frequently diagnosed cancer and the 4th highest in terms of mortality worldwide [1, 2], endoscopic submucosal dissection has emerged as a preferred minimally invasive treatment for early-stage esophageal squamous cell carcinoma, offering an improved prognosis for patients [3]. However, with disease progression, treatment complexities arise, resulting in a diminished prognosis and elevated mortality rates. The survival rate for early-stage esophageal cancer exceeds 90%, but upon advancing to later stages, it dramatically drops to less than 5% [1, 4]. These statistics underscore the pivotal role of early detection and treatment for esophageal squamous cell carcinoma. Hence, it is essential to explore the molecular mechanisms underlying the advancement of esophageal squamous cell carcinoma and identify potential targets to hinder its progression.

Cancer stem cells, characterized by their ability to self-renew and maintain a stemness state [5, 6], significantly contribute to tumor formation, maintenance, progression, and drug resistance [7]. Consequently, targeting the signaling pathways associated with these cancer stem cells emerges as a promising strategy for cancer treatment.

The insulin-like growth factor 1 receptor, a receptor tyrosine kinase [8], plays an indispensable role in activating PI3K/AKT and MEK/ERK upon binding with IGF-1 [9]. The IGF-1/IGF-1R signaling pathway is crucial for fostering proliferation and inhibiting apoptosis in various malignant diseases [10, 11]. Moreover, it is implicated in regulating cancer stem cells across different cancers, maintaining stem cell-like properties, and fostering drug resistance [12–16]. Studies suggest a strong correlation between the overexpression of IGF-1R and a poor prognosis in esophageal squamous cell carcinoma [17]. Additionally, IGF-1 is implicated in promoting the proliferation of esophageal cancer cells, as well as contributing to their drug resistance [18]. These findings underscore the pivotal role of the IGF/IGF-1R signaling pathway in the onset and progression of esophageal squamous cell carcinoma.

Forkhead box (FOX) proteins are a significant family of transcription factors, characterized by a wing-helical DNA binding domain [19]. Numerous studies have established that these proteins play a crucial role in regulating cancer stem cells in various malignant tumors. For example, FOXA2 stimulates triple-negative breast cancer cell proliferation, maintaining their stem-like properties and promoting recurrence after treatment [20]. FOXG1 maintains self-renewal ability and promotes glioblastoma tumorigenesis via the interaction with domain BMIL [21]. Additionally, FOXP1 and FOXO4 prompt the maintenance of stemness and drug resistance in ovarian cancer and diffuse large B lymphoma cells, respectively [22, 23]. As a significant member of the FOX family, FOXC1 is implicated in embryonic development and organogenesis [24]. Numerous studies have validated its crucial role in various malignancies such as breast cancer, pancreatic cancer, and hepatocellular carcinoma, demonstrating a strong association with poor prognosis [25, 26]. Recent studies establish a stronger connection between elevated FOXC1 expression and indicators of poor differentiation, metastasis, and advanced lymph node classification, underscoring the importance of FOXC1 [27]. However, the function of FOXC1 in ESCC remains poorly studied, with no reports on its regulation of cancer stem cells in this context.

Our research uncovered that IGF-1 upregulates FOXC1 expression through the IGF-1/IGF-1R/PI3K-AKT/NF-κB and IGF-1/IGF-1R/MEK/ERK/NF-κB signaling pathways. FOXC1, in turn, promotes esophageal squamous cell carcinoma stem-like properties by transactivating CBX7 and IGF-1R. This establishes a significant positive feedback loop (IGF-1/IGF-1R-FOXC1-IGF-1R), underscoring FOXC1’s potential role as a prognostic biomarker for esophageal squamous cell carcinoma. Additionally, targeting the IGF-1/IGF-1R-FOXC1-IGF-1R loop emerges as a promising therapy for anti-cancer stem cell therapy in esophageal squamous cell carcinoma.

## Results

### FOXC1 expression is upregulated in esophageal squamous cell carcinoma and negatively correlates with survival probability

**Fig. 1:**
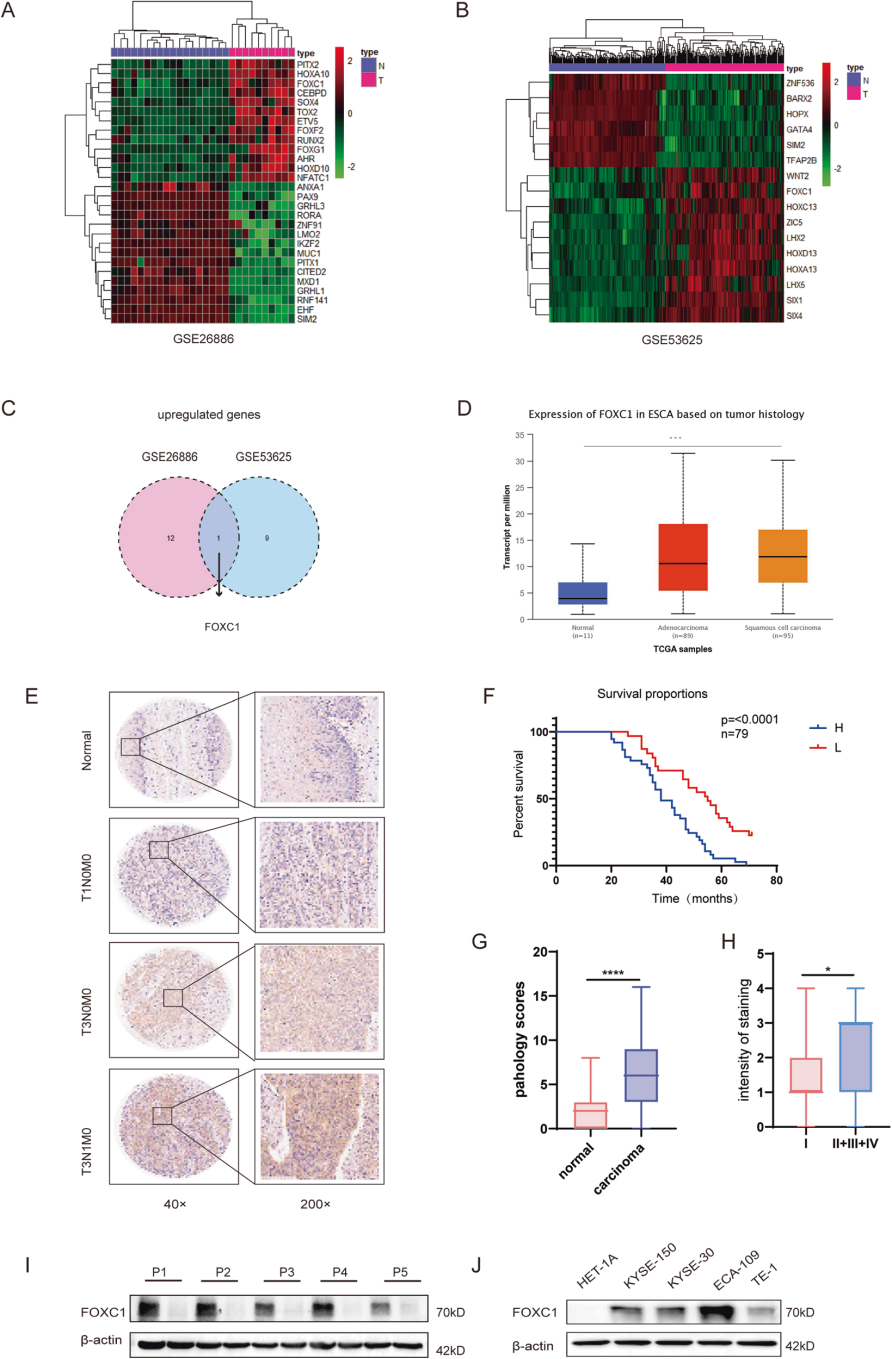
FOXC1 is highly expressed in esophageal squamous cell carcinoma and negatively correlates with survival probability. **A**, **B** Heatmap of differentially expressed genes of GSE26886 (**A**) and GSE53625 (**B**). **C** A Venn diagram illustrates the overlap of upregulated genes in both GSE26886 and GSE53625(fold change >3.0).FOXC1 is upregulated in both GSE26886 and GSE53625. **D** TCGA database shows that FOXC1 is highly expressed in ESCC tissues compared to normal esophageal tissues. **E** Esophageal squamous cell carcinoma tissue microarray from 79 patients shows that FOXC1 is highly expressed in ESCC tissues compared to normal esophageal tissues. **F** The expression level of FOXC1 in ESCC was negatively correlated with patient survival rate. **G**, **H** The expression level of FOXC1 is corresponding to ESCC progression. **I** FOXC1 expression level in ESCC was higher than that in corresponding paraneoplastic tissues of 5 randomly selected patients. **J** FOXC1 is higher in ESCC cell lines especially in KYSE-150, ECA-109, TE-1, and KYSE-30 than in normal esophageal epithelial cell line HET-1A. *P < 0.05; **P < 0.01; ***P < 0.001;****p < 0.0001.

We analyzed RNA-seq sequencing datasets (GSE53625, GSE26886) of ESCC, available on the Gene Expression Omnibus (Fig. 1A, B). Our analysis of upregulated genes led to the identification of transcription factors expressed at elevated levels in both datasets. The results revealed upregulated expression of FOXC1 in ESCC, suggesting its potential significance in ESCC progression(Fig. 1C). We further analyzed FOXC1 expression in esophageal squamous cell carcinoma, esophageal adenocarcinoma, and normal esophageal tissues based on the TCGA database. In comparison to normal esophageal tissues, the results indicated a significant increase in FOXC1 expression in esophageal squamous cell carcinoma (Fig. 1D). Furthermore, immunohistochemical staining of collected carcinoma and paraneoplastic tissues exhibited a notable increase in FOXC1 expression in ESCC. We observed a close correlation between FOXC1 expression and the progression of ESCC (Fig. 1E, G, H). FOXC1 expression in ESCC exhibited a negative correlation with patient survival rate (Fig. 1F). Additionally, western blot analysis provided further confirmation of the higher FOXC1 expression level in ESCC compared to corresponding paraneoplastic tissues (Fig. 1I). Furthermore, western blot analysis demonstrated elevated FOXC1 expression levels in esophageal squamous cell carcinoma cell lines (KYSE150, TE-1, ECA-109, KYSE-30) compared to the normal esophageal epithelial cell line HET-1A (Fig. 1J).

### FOXC1 enhances the stemness of ESCC cells in vitro

**Fig. 2:**
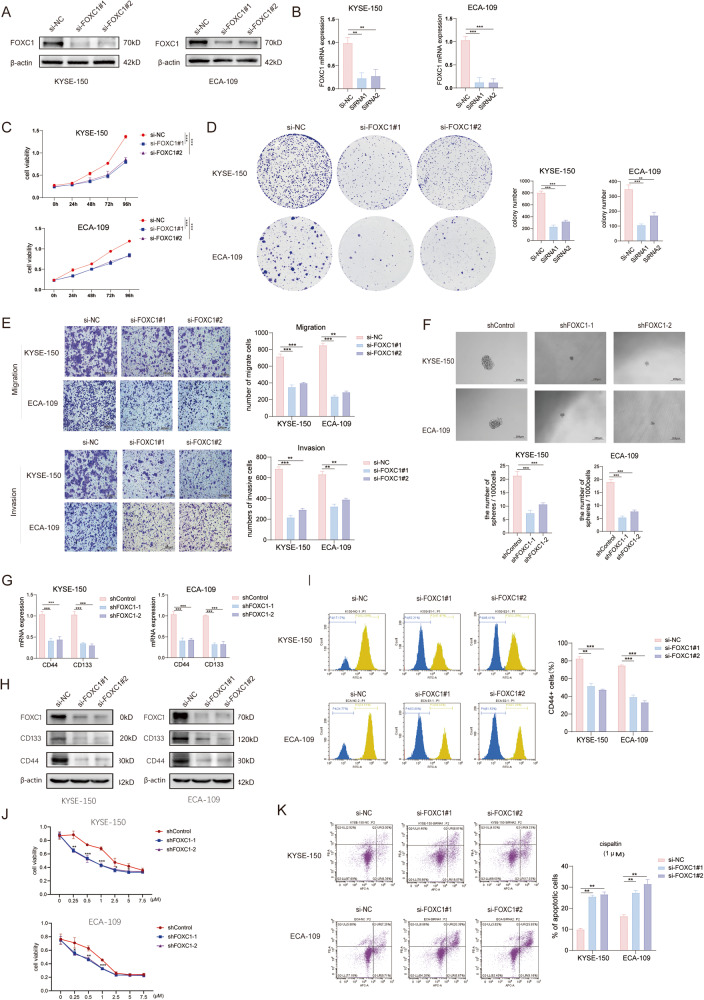
FOXC1 enhances stemness of ESCC cells in vitro. **A**, **B** Western blot analysis(A) and qPCR analysis(B) indicated that FOXC1 was significantly inhibited by siRNA in KYSE-150 and ECA-109 cells. (si-NC: si-Negative Control). **C** FOXC1 knockdown markedly inhibited the proliferation ability of ECA-109 and KYSE-150 cells indicated by CCK8 assay. **D** FOXC1 knockdown markedly inhibited the proliferation ability of ECA-109 and KYSE-150 cells indicated by colony formation assay. **E** FOXC1 knockdown markedly inhibited the invasion and migration ability of ECA-109 and KYSE-150 cells indicated by transwell assay. The scale bars represent 200μm. **F** FOXC1 knockdown markedly inhibited the sphere-formation ability of ECA-109 and KYSE-150 cells. The scale bars represent 200μm. **G** QPCR analysis indicated that FOXC1 knockdown markedly downregulated mRNA expression of cancer stem cell markers CD44 and CD133 in ECA-109 and KYSE-150 cells. **H** Western blot analysis indicated that FOXC1 knockdown markedly downregulated protein expression of cancer stem cell markers CD44 and CD133 in ECA-109 and KYSE-150 cells. **I** FACS indicated that FOXC1 knockdown markedly decreased the percentage of CD44+ cells. **J**, **K** Cell viability was detected after cells were treated with cisplatin at the indicated concentrations(1 μM) for 24 h by CCK8 assay (**J**) and apoptosis assay (**K**). *P < 0.05; **P < 0.01; ***P < 0.001; ****P < 0.0001.The *p* values were obtained by two-way ANOVA (**C**, **J**) or one-way ANOVA (others). Data are presented as mean (*n* = 3) ± S.D. All experiments were performed at least three times.

The stem cell-like properties significantly enhanced the in vitro proliferation, motility, tumorigenesis, and drug resistance of tumor cells [28]. SiRNA was employed to downregulate FOXC1 expression in ECA-109 and KYSE-150 cell lines, and the efficacy was confirmed through western blot and RT-qPCR analysis (Fig. 2A, B). The CCK8 assay (Fig. 2C) and colony formation assay (Fig. 2D) revealed a substantial reduction in the proliferation capability of tumor cells following FOXC1 knockdown. Transwell experiments demonstrated a significant reduction in the invasion and migration capabilities of ECA-109 and KYSE-150 cells after FOXC1 knockdown (Fig. 2E). Furthermore, FOXC1 knockdown led to a notable reduction in the in vitro sphere-forming ability of tumor cells (Fig. 2F). In addition, employing western blot and RT-qPCR analysis, our investigation demonstrated a substantial decline in the levels of cancer stem cell markers CD133 and CD44 within the knockdown groups. (Fig. 2G, H). FACS analysis revealed a significant decrease in the proportion of CD44+ cells (Fig. 2I). Simultaneously, IHC staining results illustrated a positive correlation between FOXC1 expression and the expression of CD133 and CD44 (Fig. S1A–C). The stem-like properties are also crucial contributors to promoting drug resistance [28]. Through the CCK8 assay (Fig. 2J) and apoptosis assay (Fig. 2K), we observed heightened sensitivity of tumor cells to cisplatin following FOXC1 knockdown.

### FOXC1 enhances the tumorigenicity of ESCC cells in vivo

**Fig. 3:**
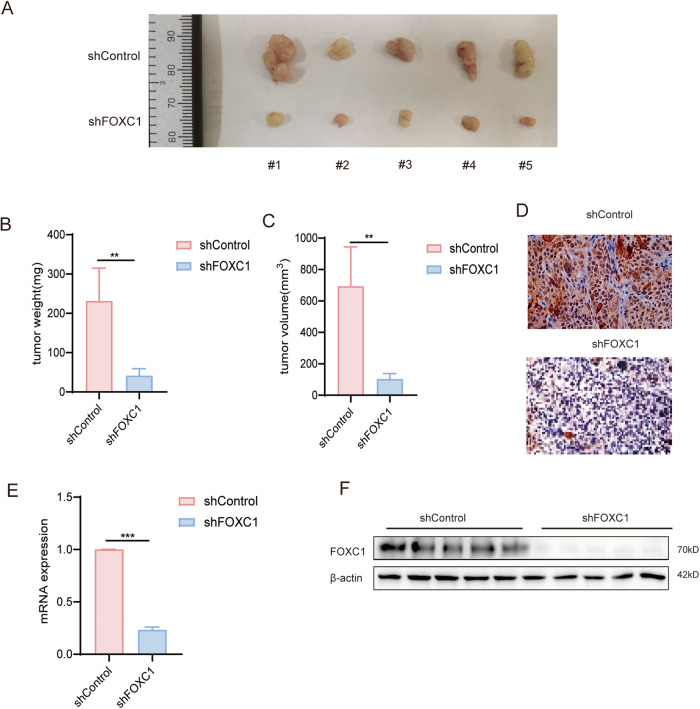
FOXC1 enhances tumorigenicity of ESCC cells in vivo. **A** The tumor incidence of different groups, including shFOXC1 or negative control group. (*n* = 5/group). **B** FOXC1-knockout group had significantly lower tumor weight compared to the control group. **C** FOXC1-knockout group had significantly lower tumor volume compared to the control group. **D–F** Western blot analysis, qPCR analysis, and IHC indicated that FOXC1 was significantly inhibited by Lv-shFOXC1 in KYSE-150 cells. *P < 0.05; **P < 0.01; ***P < 0.001; ****P < 0.0001.The *p* values were obtained by *t* test (**B**, **C**, **E**). Data are presented as mean (*n* = 3) ± S.D. All experiments were performed at least three times.

We inoculated the same amount of KYSE-150-shFOXC1-LV and KYSE-150-shControl-LV cell lines subcutaneously into the right abdomen of two groups of 5-week-old BALB/c nude mice. The mice were euthanized, and the tumors were subsequently extracted after 4 weeks(Fig. 3A). We then measured and analyzed the weight and volume of each tumor. Subsequent measurements and analyses of the weight and volume of each tumor indicated that the FOXC1-knockout group exhibited significantly lower tumor volume and weight compared to the control group (Fig. 3B, C). The expression of FOXC1 was verified by RT-qPCR, western blot analysis, and immunohistochemistry (Fig. 3D–F). These findings provide compelling evidence that FOXC1, as a transcription factor, plays a pivotal role in promoting stem-cell properties and tumorigenicity in vivo.

### FOXC1 affects the stemness of esophageal squamous cell carcinoma by regulating CBX7 and IGF-1R

**Fig. 4:**
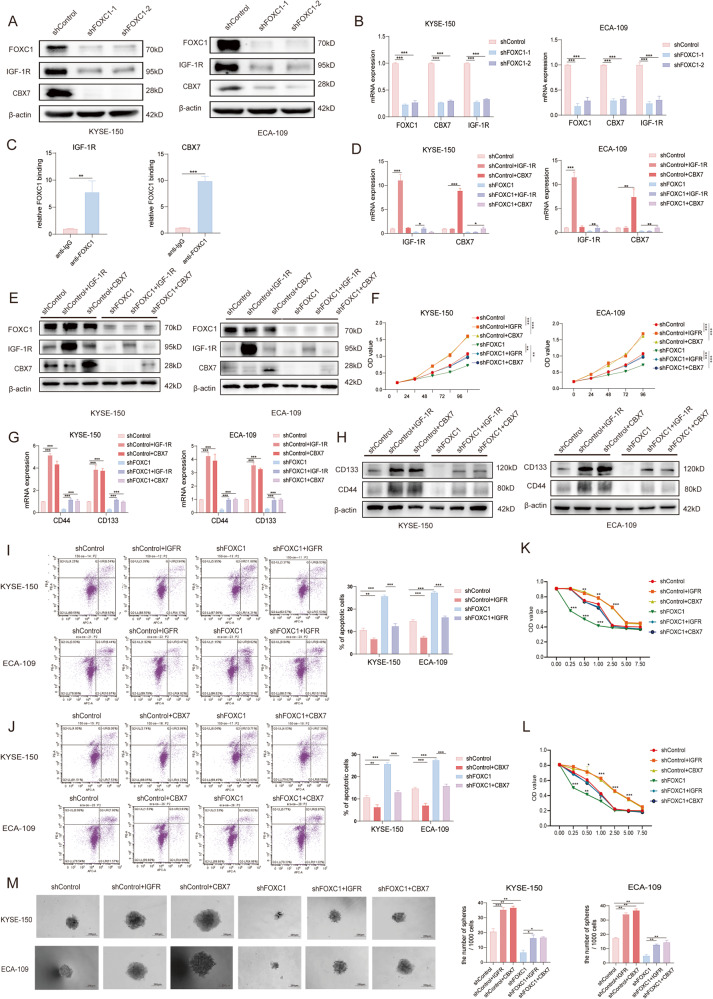
FOXC1 affects the stemness of esophageal squamous cell carcinoma by regulating CBX7 and IGF-1R. **A** Western blot analysis indicated that the knockdown of FOXC1 could downregulate CBX7 and IGF-1R expression in ECA-109 and KYSE-150 cells. **B** QPCR analysis indicated that the knockdown of FOXC1 could downregulate CBX7 and IGF-1R mRNA expression in ECA-109 and KYSE-150 cells. **C** ChIP assays revealed the direct binding of FOXC1 to the CBX7(right) or IGF-1R (left) promoter in KYSE-150 cells. **D**, **E** The mRNA expression of FOXC1, IGF-1R, and CBX7 was analyzed by qPCR(D), and The protein level of FOXC1, IGF-1R, and CBX7 was analyzed by western blot (**E**) after conducting transfection experiments using plasmids overexpressing CBX7 and IGF-1R in KYSE-150-shControl-LV, KYSE-150-shFOXC1-LV, ECA-109-shControl-LV, and ECA-109-shFOXC1-LV cell lines. **F** CCK8 assay indicated that the overexpression of CBX7 and IGF-1R significantly increased the proliferation ability of tumor cells, and rescued the negative impact of FOXC1 knockdown on tumor cell proliferation. **G**, **H** QPCR analysis (**G**) and western blot analysis (**H**) indicated that overexpressing CBX7 or IGF-1R could upregulate the expression of cancer stem cell marker CD44 and CD133 in ECA-109 and KYSE-150 cells and rescue the negative influence of FOXC1 knockdown. I-L.Upregulation of CBX7 and IGF-1R enhanced the resistance of KYSE-150 and ECA-109 cells to cisplatin through apoptosis assay (**I**, **J**) and CCK8 assay (**K**, **L**). **M** Upregulation of CBX7 and IGF-1R promoted the sphere-forming ability and rescued the influence of FOXC1 knockdown. The scale bars represent 200μm. *P < 0.05; **P < 0.01; ***P < 0.001; ****P < 0.0001. The *p* values were obtained by *t* test (**C**), two-way ANOVA (**F**, **K**), or one-way ANOVA (others). Data are presented as mean (*n* = 3) ± S.D. All experiments were performed at least three times.

To elucidate the impact of FOXC1 on stem-like properties in ESCC, we employed siRNA to knock down FOXC1 in ECA-109 and KYSE-150 cell lines, subsequently conducting RNA sequencing to analyze differentially expressed genes. Using a 3-fold cut-off, we identified 62 in ECA-109-siFOXC1 cells and 260 down-regulated genes in KYSE-150-siFOXC1 cells. Intersection analysis pinpointed four genes with consistently reduced expression, notably observing significant down-regulation of CBX7 and IGF-1R upon FOXC1 knockdown (Figure S1D). Lentivirus vectors were utilized for FOXC1 knockdown in ECA-109 and KYSE-150 cells, with confirmation of CBX7 and IGF-1R down-regulation through western blot analysis(Fig. 4A) and RT-qPCR analysis(Fig. 4B). A ChIP assay validated the direct binding of FOXC1 to the promoter region of CBX7 and IGF-1R (Fig. 4C). To explore the involvement of CBX7 and IGF-1R in FOXC1-mediated stemness regulation in ESCC, we conducted transfection experiments using plasmids overexpressing CBX7 and IGF-1R in KYSE-150-shControl-LV, KYSE-150-shFOXC1-LV, ECA-109-shControl-LV, and ECA-109-shFOXC1-LV cell lines. Transfection efficiency was assessed by western blot and RT-qPCR analysis (Fig. 4D, E). CCK8 assays revealed that CBX7 and IGF-1R overexpression significantly increased tumor cell proliferation, counteracting the negative impact of FOXC1 knockdown (Fig. 4F). Additionally, western blot and RT-qPCR analysis confirmed the positive regulation of CBX7 and IGF-1R on cancer stem cell markers CD133 and CD44, rescuing the negative influence of FOXC1 knockdown on these markers (Fig. 4G, H). Moreover, upregulation of CBX7 and IGF-1R enhanced the resistance of ECA-109 and KYSE-150 cells to cisplatin, as demonstrated by apoptosis assay (Fig. 4I, J) and CCK8 assays (Fig. 4K, L). Notably, in KYSE-150-shFOXC1-LV and ECA-109-shFOXC1-LV cells, the overexpression of CBX7 and IGF-1R counteracted the negative effects induced by FOXC1 knockdown. In the sphere-forming experiment, a similar effect was observed, with the upregulation of CBX7 and IGF-1R promoting sphere-forming ability and rescuing the influence of FOXC1 knockdown (Fig. 4M). Collectively, these findings affirm the significant role of CBX7 and IGF-1R in FOXC1-induced stem cell-like properties in ESCC.

### FOXC1 is critical for IGF-1-induced ESCC stemness

**Fig. 5:**
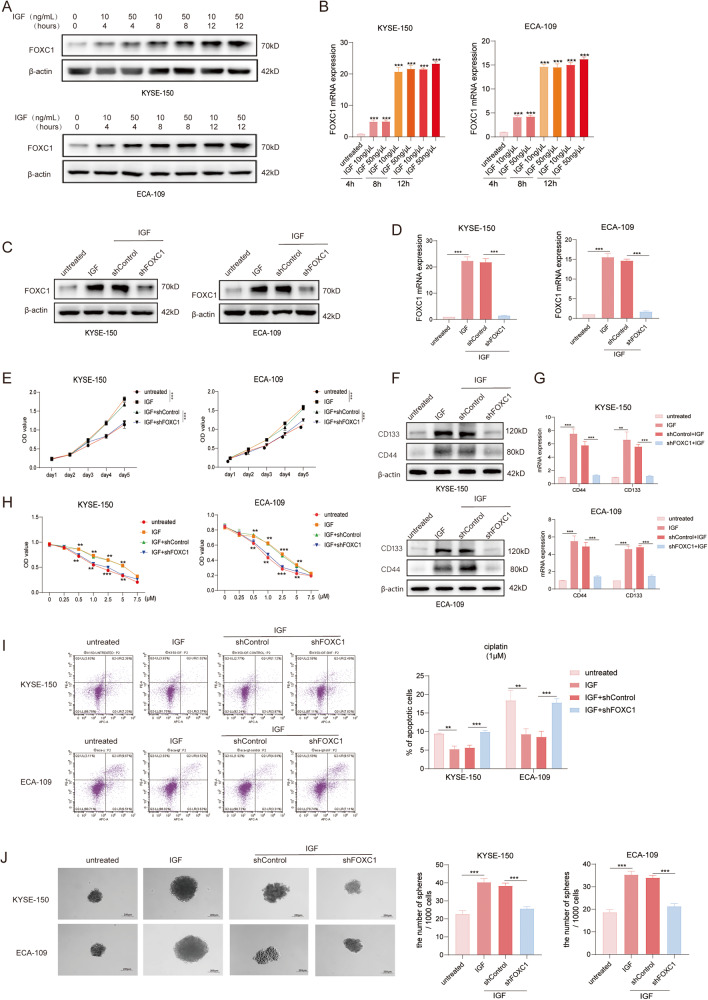
FOXC1 is critical for IGF-1-induced ESCC stemness. **A**, **B** KYSE-150 and ECA-109 cells were administrated with 10 ng/mL、50 ng/mL IGF-1 for 0, 4, 8, 12 h, and then the mRNA and protein levels of FOXC1 were examined by western blot analysis (**A**) and qPCR analysis (**B**). **C**, **D** KYSE-150 and ECA-109 cells were transfected with LV-shcontrol or LV-shFOXC1 lentiviral vectors and then treated with or without IGF-1 (50 ng/mL, 12 h). Next, the FOXC1 expression was analyzed by western blot analysis (**C**) and qPCR analysis (**D**). **E** CCK8 assay demonstrated that IGF-1 treatment increased the proliferation ability of KYSE-150 and ECA-109 cells, while FOXC1 knockdown reduced these capabilities. **F**, **G** Western blot analysis (**F**) and qPCR analysis (**G**) indicated that IGF-1 treatment increased the expression of cancer stem cell markers CD44 and CD133 while FOXC1 knockdown reduced the expression of CD44 and CD133. **H**, **I** IGF-1 treatment increased the tolerance to cisplatin while FOXC1 knockdown reduced the effect. The cell viability was detected by CCK8 assay (**H**) and apoptosis assay (**I**). **J** Sphere-formation assay indicated that IGF-1 treatment increased the sphere-formation ability of KYSE-150 and ECA-109 cells, while FOXC1 knockdown reduced these capabilities. The scale bars represent 200μm. *P < 0.05; **P < 0.01; ***P < 0.001; ****P < 0.0001.The *p* values were obtained by two-way ANOVA (**E**, **H**) or one-way ANOVA (others). Data are presented as mean (*n* = 3) ± S.D. All experiments were performed at least three times.

**Fig. 6:**
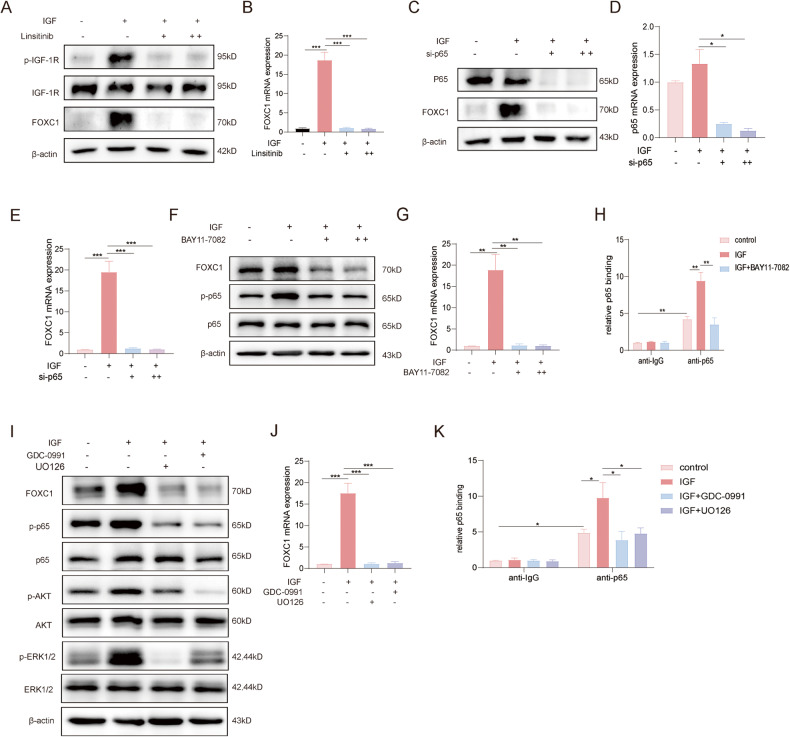
IGF-1-IGF-1R signaling upregulates FOXC1 expression via PI3K/AKT/NF-κB; ERK/NF-κB pathway. **A**, **B** KYSE-150 cells were treated with IGF-1(50 ng/mL) for 12 h in the presence or absence of 10 μM、20 μM IGF-1R inhibitor Linsitinib, the protein expression of FOXC1, phosphorylated and total IGF-1R was detected by western blot analysis (**A**) and the mRNA expression of FOXC1 was detected by qPCR analysis (**B**). C-E.KYSE-150 cells were transiently transfected with control or p65 siRNA for 48 h and then treated with IGF-1(50 ng/mL) for 12 h. The protein level of p65, FOXC1 was detected by western blot analysis (**C**) and the mRNA of p65(**D**),FOXC1 (**E**) was detected by qPCR analysis. **F**, **G** KYSE-150 cells were treated with IGF-1(50 ng/mL) for 12 h in the presence or absence of 10 μM、20 μM BAY11-7082, the protein expression of FOXC1, phosphorylated and total p65 was detected by western blot analysis (**F**) and the mRNA expression of FOXC1 was detected by qPCR analysis (**G**). **H** The ChIP assay showed the direct binding abilities of p65 to the FOXC1 promoter induced by IGF-1, and p65 inhibitor BAY11-7082 reduced the binding abilities of p65 to the FOXC1 promoter. **I**, **J** KYSE-150 cells were treated with IGF-1(50 ng/mL) for 12 h in the presence or absence of 10 μM GDC-0991,10 μM UO126, the protein expression of FOXC1,phosphorylated and total p65, phosphorylated and total Akt and ERK1/2 was detected by western blot analysis (**I**) and the mRNA expression of FOXC1 was detected by qPCR analysis (**J**). **K** The ChIP assay showed the direct binding abilities of p65 to the FOXC1 promoter induced by IGF-1, and PI3K inhibitor and ERK inhibitor reduced the binding abilities of p65 to the FOXC1 promoter. *P < 0.05; **P < 0.01; ***P < 0.001; ****P < 0.0001. The *p* values were obtained by one-way ANOVA (**B**, **D**, **E**, **G**, **H**, **J**, **K**,). Data are presented as mean (*n* = 3) ± S.D. All experiments were performed at least three times.

Previous studies have documented the involvement of the IGF-1/IGF-1R signaling pathway in regulating cancer stem cells across various tumors, sustaining stem cell-like properties, and fostering drug resistance [15]. a ligand for IGF-1R, IGF-1 has demonstrated the ability to stimulate proliferation and enhance drug resistance in ESCC [18]. Hence, we posit a crosstalk between the IGF/IGF-1R signaling pathway and FOXC1, postulating that this pathway regulates FOXC1 expression and its capacity to sustain stemness in ESCC. Following a 12-hour treatment with IGF-1, we observed an upregulation in both FOXC1 protein and RNA expression (Fig. 5A, B). This increase was directly proportional to both the concentration of IGF-1 and the duration of treatment. ECA-109, KYSE-150, ECA-109, and KYSE-150 cells transfected with LV-shFOXC1 and LV-shControl were treated with IGF-1 (50 ng/mL, 12 h). The protein and RNA expression levels of FOXC1 were validated through western blot and RT-qPCR, respectively (Fig. 5C, D). We validated that IGF-1 enhanced the proliferative ability of tumor cells (Fig. 5E), elevated the protein and mRNA expression of cancer stem cell markers CD133 and CD44 (Fig. 5F, G), along with enhanced tolerance to cisplatinoid drugs (Fig. 6H, I) and in vitro sphere formation ability(Fig. 5J). Nevertheless, the knockdown of FOXC1 significantly mitigated these effects of IGF-1 on tumor cells.

### IGF-1-IGF-1R signaling upregulates FOXC1 expression via PI3K/AKT/NF-κB；ERK/NF-κB pathway

The activation of the IGF-1/IGF-1R signaling pathway has been shown to promote the expression of FOXC1. Our research illustrated that employing IGF-1R inhibitor Linsitinib resulted in decreased levels of both FOXC1 protein and mRNA expression., providing additional evidence to support our earlier findings (Fig. 6A, B). We further investigated the specific mechanism by which IGF-1/IGF-1R regulates FOXC1. NF-κB serves as a pivotal transcription factor with essential functions in processes such as inflammation, immune response, cellular proliferation, and apoptosis [29]. Previous studies have indicated high NF-κB expression in triple-negative breast cancer, where it promotes tumor proliferation and migration by binding to the FOXC1 promoter region, thereby increasing FOXC1 expression [30]. Moreover, NF-κB activation is facilitated by post-translational modification of NF-κB subunits by specific protein kinases, such as the phosphorylation of p65 [29, 31]. Phosphorylation modification promotes NF-κB activation and regulates gene expression. Knockdown of p65 in ECA-109 and KYSE-150 cell lines resulted in decreased protein and mRNA expression of FOXC1 (Fig. 6C–E). The use of the p65 inhibitor BAY11-7082 also downregulated FOXC1 expression at both protein and RNA levels (Fig. 6F, G). Our ChIP assay confirmed that IGF-1 promotes the phosphorylation of p65, facilitating its binding to the FOXC1 promoter. On the contrary, the p65 inhibitor BAY11-7082 significantly impeded the interaction between p65 and the FOXC1 promoter. (Fig. 6H). The PI3K/AKT and MEK/ERK pathways are the two classical cascades following IGF-1/IGF-1R signaling activation. Through the use of ERK and AKT inhibitors, we observed reduced p65 phosphorylation levels in both cases (Fig. 6I). Additionally, FOXC1 mRNA and protein expression were decreased (Fig. 6I, K). Our ChIP assay further demonstrated that both AKT and ERK inhibitors reduced the interaction between p65 and the FOXC1 promoter, confirming that IGF can enhance the phosphorylation level of p65, leading to increased binding to the FOXC1 promoter through the activation of the PI3K/AKT and MEK/ERK cascades (Fig. 6K).

## Discussion

As a highly lethal malignancy, esophageal cancer is predominantly managed via surgical, radiative, and chemotherapeutic interventions. Early-stage tumors are amenable to endoscopic resection and preoperative or perioperative chemotherapy, while advanced or metastatic cases necessitate systemic cisplatin and fluoropyrimidine (5-fluorouracil or capecitabine) regimens for palliative care [32, 33]. Despite modest enhancements in patient prognosis through conventional approaches, they remain insufficient. Cancer Stem Cells (CSCs) play an important role in both tumorigenesis and drug resistance. Exploring the mechanism of stemness transformation of ESCC tumor cells and uncovering key pathways and molecules that instigate stemness in ESCC could unveil potential therapeutic targets and herald novel breakthroughs in ESCC management.

Elevated FOXC1 expression was observed in esophageal cancer tissues, and it displayed a negative correlation with the survival rates of patients., thereby intimating FOXC1’s role as a dismal prognostic determinant in ESCC. In our current study, we unveil for the first time that FOXC1 participates in ESCC stemness regulation. CD133 and CD44 are classical cancer stemness markers of ESCC [34, 35]. We discovered that the knockdown of FOXC1 significantly reduced the expression of CD44, and CD133 at the protein and mRNA levels, subsequently curtailing the proportion of CD44+ cells within esophageal cancer cell lines ECA-109 and KYSE-150. Additionally, sphere-formation capability, emblematic of self-renewal—one of CSC’s pivotal traits—markedly dwindles in the FOXC1 knockdown group, signified by markedly smaller spheres in contrast to the control group.

In addition, stemness imparts an influence on tumor cell proliferation, both in vitro and in vivo. FOXC1 knockdown engenders a decelerated growth rate within the FOXC1 knockdown group, evident from the CCK8 assay, and corroborated by tumor size and weight differentials in the in vivo comparison with the control group. These compellingly confirm FOXC1’s pivotal role in maintaining stem cell-like attributes within tumors.

Drug resistance stands as a pivotal challenge in ESCC treatment, constituting a hallmark feature of CSCs. Though chemotherapeutic agents target tumor cells, CSCs demonstrate resilience to chemotherapy, culminating in tumor recurrence [36]. Cisplatin, a cornerstone of ESCC chemotherapy, grapples with drug resistance challenges. FOXC1 knockdown augments tumor cells’ susceptibility to cisplatin, implying FOXC1’s potential utility as a bioindicator and resistance target in esophageal cancer treatment.

The polycomb protein family PcG is a class of transcription factors that orchestrates gene expression at the chromatin level through epigenetic modifications, mainly in the form of polycomb repressor complexes 1 and 2 (PRC1 and PRC2) [37]. PcG proteins act at different loci of chromosomes by forming polycomb repression complexes (PRCs), which modify chromosome structure and suppress gene expression [37]. During the differentiation of pluripotent embryonic stem cells (ESCs), CBX proteins (CBX2/4/6/7/8), as components of the canonical PRC1 complex, play a dynamic role in maintaining a balance between self-renewal and differentiation [38]. CBX7 embraces multifaceted roles across distinct tumor types, involving its downregulation in renal clear cell carcinoma and its association with favorable prognoses, suppression of BLBC growth and metastasis via the Twist1/EphA2 pathway, and a paradoxical involvement in bladder cancer, wherein it dampens FGFR3 expression to attenuate cisplatin sensitivity [39, 40]. However, the pro-carcinogenic role of CBX7 in tumors has also been extensively studied. In ovarian clear cell adenocarcinoma, expression of chromobox homolog 7 (CBX7) portends an ominous prognosis [41]. Via the AKT-NF-κB-miR-21 and p16 pathways, CBX7 exerts control over the stem cell-like properties of cancer cells [42]. CBX7, chiefly entrusted with sustaining ESC and hematopoietic stem cell (HSC) pluripotency, achieves this feat through heightened expression in stem cells, quelling spectrum-specific gene activity, and forestalling premature differentiation [43]. These connote the intimate correlation of CBX7 with cell pluripotency and self-renewal capacity. Based on the sequencing results and the fact that CBX7 regulates stem cell differentiation and renewal, we hypothesized that CBX7 promotes tumor development and drug resistance by impacting ESCC stemness.

As previously stated, the IGF-1/IGF-1R signaling pathway mediates the development of diverse tumors and is involved in maintaining CSCs and drug resistance. IGF-1R’s ominous association with ESCC prognosis underscores its promise as a therapeutic target. In this study, we found that overexpression of CBX and IGF-1R, respectively, resulted in a remarkable increase in sphere-formation capacity, proliferation ability, and drug resistance of tumor cells. These confirmed the role of CBX7 and IGF-1R for stemness induction in ESCC. In addition, we demonstrated that FOXC1 transactivated CBX7 and IGF-1R, and overexpression of CBX7 and IGF-1R rescued the negative effect of FOXC1 knockdown on ESCC stemness. Thus, we conclude that FOXC1 promotes the stem-cell-like properties of ESCC through the upregulation of CBX7 and IGF-1R.

Subsequently, we explored the molecular mechanisms propelling FOXC1-triggered CSC-like properties in ESCC. IGF-1R, a pivotal receptor tyrosine kinase, garners significance as a therapeutic focus across myriad tumor interventions. The ligand IGF-1 has a high affinity for IGF-1R. Multiple studies corroborate the pivotal role of the IGF-1/IGF-1R signaling pathway in tumor progression and drug resistance. Concurrently, some reports also confirmed the high expression of IGF-1R in ESCC and the promotion of proliferation and drug resistance of IGF-1 in esophageal cancer cells [17, 18]. Our study exposes the IGF-IGF-1R cascade’s capacity to amplify FOXC1 expression by eliciting MEK/ERK/NF-κB and PI3K/AKT/NF-κB pathways. This prompts FOXC1 upregulation and, in turn, potentiates IGF-1R expression, establishing a positive feedback loop—IGF-1-FOXC1-IGF-1R. Furthermore, we demonstrated that the knockdown of FOXC1 inhibited IGF-1-induced ESCC stemness. Thus, such a positive feedback pathway may inspire more novel ESCC treatments.

In summary, our findings illuminate that IGF-1 mediates FOXC1 expression, while FOXC1 upregulates CBX7 and IGF-1R expression and induces ESCC stemness. Thus, FOXC1 emerges as a potential prognostic indicator, while the IGF-1-FOXC1-IGF-1R oncogenic loop stands out as a prospective therapeutic avenue for mitigating FOXC1-induced ESCC stemness.

## Methods and materials

### Data collection

A comprehensive cancer genomics program, The Cancer Genome Atlas (TCGA) has conducted molecular characterizations of 33 primary cancer types. Using UALCAN (https://ualcan.path.uab.edu/analysis.html), exploration of FOXC1 expression in esophageal squamous cell carcinoma was conducted utilizing data extracted from the TCGA database.

### Cell lines and culture conditions

Human esophageal squamous cell carcinoma cell lines, such as TE-1, ECA-109, KYSE-30, and KYSE-150, were procured from the Institute of Biological Sciences of the Chinese Academy of Sciences in Shanghai. Subsequently, routine mycoplasma contamination testing was conducted. Cells were maintained at 37°C with 5% CO2, cultured in DMEM medium (GIBCO) containing 1% penicillin-streptomycin and 10% Fetal Bovine Serum (GIBCO).

### Transient transfection and establishment of stable cell lines

Cells were seeded into 6-well plates for transient knockdown transfection, followed by the transfection of 100pmol siRNA-FOXC1 (GenePharma, Shanghai, China) using HighGene (ABclonal, Wuhan, China) ECAh well, following the manufacturer’s guidance. Cells were seeded into 6-well plates for transient gene overexpression transfection, followed by the transfection of the plasmids expressing CBX7 or IGF-1R were purchased from Genechem (Shanghai, China) using HighGene (ABclonal, Wuhan, China).

At 48 h post-transfection, transfection efficiency was assessed using RT-qPCR and western blot. For stable transfection, ECA-109 and KYSE-150 cells were transfected with lentivirus vectors encoding either FOXC1-targeting shRNA or non-targeting control shRNA, following the manufacturer’s instructions (Genechem, Shanghai, China). Briefly, cells were seeded in 6-well plates, and when the cell density reached 30%, a medium containing viral fluid at an MOI of 10, without serum, was added. This medium was replaced with a complete medium 24 h later. After lentiviral infection, ECA-109 cells and KYSE-150 cells underwent a two-week selection process with 1 µg/mL puromycin to obtain stable clones. Transfection efficiency for each vector was evaluated through a western blot.

### Cell invasion and migration assays

In the cell migration experiment, 1 × 10^5^ cells were re-suspended in 200 μL serum-free DMEM medium and added to the upper compartment, and 500 l DMEM medium containing 10%FBS was added to the lower compartment to induce the migration of cells. In the cell invasion experiment, the cells re-suspended in 200 μL serum-free DMEM medium were added to the upper chamber coated with Matrigel matrix (Corning, 356234), and the rest procedures were performed the same as the cell migration experiment. Fixation with 4% paraformaldehyde and staining with crystal violet dye were conducted after a 24-h incubation period. Subsequently, IMAGEJ software was employed for cell number quantification.

### Cell counting Kit-8 (CCK-8) proliferation assay

We introduced a seeding density of 2000 cells per well into 96-well plates and established an arrangement of 10 sub-wells. After the cells were fully attached to the plate, CCK8 reagent (10 μl per well) was added at 0,24,48,72,96 h, respectively. Subjected to incubation in the absence of light for an hour, the microplate reader was employed to analyze the absorbance at 450 nM. Three repetitions of the experiments were executed, followed by the final statistical analysis performed using GraphPad Prism 8.0.

### Colony formation assay

6-well plates were used for cell inoculation, with ECAh well receiving 1 × 10^3^ cells, and subsequent culture was carried out in DMEM medium containing 10% FBS and 1% Penicillin-Streptomycin Solution. After a 14-day incubation period, cell fixation was performed using 4% paraformaldehyde, followed by staining with 0.1% crystal violet dye. The colony count was determined using ImageJ software.

### Sphere-formation analysis

The ECA-109 cells and KYSE-150 cells were plated into ultra-low six-well plates (Corning) at 1 × 10^3^ cells/well. The cells were cultured in serum-free DMEM/F12(Gibco) with 2% B27(Invitrogen)、20 ng/mL EGF(PeproTech)、20 ng/mL bFGF(PeproTech) for 14 days. The size of the tumor spheroids was observed under a light microscope and the count of spheres with a diameter greater than 100 μM was counted.

### Apoptosis assay

ECA-109 cells and KYSE-150 cells were incubated in 6-well plates in DMEM supplemented with 10% FBS, 1% penicillin-streptomycin, and 1 μM cisplatin. After incubation for 24 h, the cells were gathered, and an Annexin V-FITC apoptosis analysis kit (Elabscience Biotechnology) was utilized to assess the percentage of apoptotic cells, following the step-by-step instructions in the user manual. Results were represented as the mean of % cell death of at least three independent replicates.

### Flow cytometric analysis

1 × 10^6^ ECA-109 cells and 1 × 10^6^ KYSE-150 cells were incubated with CD44 antibody(R&D Systems)for 10 min at room temperature, and washed twice twice after that. The FACS was performed using the Beckman CytoFLEX and the percentage of CD44+ cells was analyzed.

### RT-qPCR assay, and RNA sequencing

Cells were subjected to RNA isolation using Trizol (Vazyme) followed by reverse transcription into cDNA using the Reverse Transcriptase Kit (Abclonal). RT-qPCR was performed with the primers for FOXC1, CBX7, IGF- 1 R, CD133, CD44, and β-actin, and the fold change was calculated by the 2-ΔΔCt method. Cloud-Seq Biotech (Shanghai, China) conducted RNA high-throughput sequencing, wherein the removal of rRNAs was accomplished using the GenSeq® rRNA Removal Kit (GenSeq, Inc.) with total RNA. After the removal of rRNA from the samples, library construction was carried out utilizing the GenSeq® Low Input RNA Library Prep Kit (GenSeq, Inc.), following the prescribed protocol from the manufacturer. Quality control and quantification of the libraries were executed using the BioAnalyzer 2100 system (Agilent Technologies, Inc., USA). The sequencing of the libraries transpired on an Illumina Novaseq instrument, employing 150 bp paired-end reads. Primer sequences are listed in Table 1.

**Table 1:** Sequences of each assay.

assay	name	sequence
siRNA	si-NC-sense	5’-UUCUCCGAACGUGUCACGUTT-3’
	si-NC-antisense	5’-ACGUGACACGUUCGGAGAATT-3’
	si-FOXC1#1-sense	5’-GCAUCUACCAGUUCAUCAUTT-3’
	si-FOXC1#1-antisense	5’-AUGAUGAACUGGUAGAUGCTT-3’
	si-FOXC1#2-sense	5’-CCUACAACAUGUUCGAGAATT-3’
	si-FOXC1#2-antisense	5’-UUCUCGAACAUGUUGUAGGTT-3’
RT-qPCR	FOXC1-F	5’-TCACAGAGGATCGGCTTGAACAAC-3’
	FOXC1-R	5’-ACAGAGACTGGCTGGAAGGGAAG-3’
	CBX7-F	5’-GCGTGCGGAAGGGTAAAGT-3’
	CBX7-R	5’-GCTTGGGTTTCGGACCTCTC-3’
	IGF-1R-F	5’-CTGCTTAGCTTCCTTGCCTCC-3’
	IGF-1R-R	5’-TTATAAACACGCCACGGCCC-3’
	CD44-F	5’-AGCCCATGTTGTAGCAAACC-3’
	CD44-R	5’-TGAGGTACAGGCCCTCTGAT-3’
	CD133-F	5’-AATGCACCAGCGACAGAAG-3’
	CD133-R	5’-CATTCAAGAGAGTTCGCAAGTC-3’
Chromatin immunoprecipitation	FOXC1-F1	5’-AATCTCAGGCGTGTGCACAA-3’
	FOXC1-R1	5’-CTAAGAGCTTAACGCTTCCC-3’
	CBX7-F1	5’-GAGAAACCCACAAGAGACAC-3’
	CBX7-R1	5’-ACTGCACCAGCCTTCTTTTC-3’
	IGF-1R-F1	5’-ACCTCTCCTCCTGCACATTC-3’
	IGF-1R-R1	5’-TCTCCGCGGTTGTTTTCTTG-3’

### Western blot

Proteins were extracted using RIPA lysate (Beyotime) supplemented with 1% PMSF (Beyotime) and 2% phosphatase inhibitor (Beyotime). Following electrophoretic separation through SDS-PAGE, the proteins were transferred onto PVDF membranes. After blocking with 5% skim milk, primary antibodies specific for FOXC1 (ab227977, Abcam,1:1000), CD44 (A19020, Abclonal,1:1000), CD133 (A0219, Abclonal,1:1000), CBX7 (ab178411, Abcam,1:1000), IGF-1R (ab182408, Abcam,1:1000), phosphor-IGF-1R (ab39398, Abcam,1:1000), Akt (4691, Cell Signaling Technology,1:1000), phospho-Akt (S473) (4060, Cell Signaling Technology,1:1000), ERK1/2 (ab184699, Abcam,1:1000), phospho -ERK1/2 (ab201015, Abcam,1:1000) primary antibodies overnight and β-actin (AC026, Abclonal,1:10000) as internal reference were used for protein examination. .

### Chromatin immunoprecipitation assay

Cultivated cells were fixed, chromatin sonicated, immunoprecipitated, and DNA purified according to ChIP-IT High Sensitivity kit (Active Motif) instructions, and the relative abundance of target DNA was analyzed by qPCR. Primer sequences are listed in Table 1.

### Immunohistochemistry

Tissue samples for this study were sourced from individuals diagnosed with esophageal squamous cell carcinoma at Tongji University’s Dongfang Hospital, totaling 79 patients. Following fixation in formalin and embedding in paraffin, tissue sections were sliced to a thickness of 4μm. Subsequently, the sections underwent deparaffinization and hydration through immersion in xylene and graded alcohols. Heat-induced antigen retrieval was conducted in EDTA buffer (pH 8.0) for 15 minutes, utilizing a microwave oven. To minimize nonspecific staining, blocking was carried out with 10% goat serum. Following this, specific primary antibodies, including FOXC1 (ab227977, Abcam, 1:200), CD44 (A19020, Abclonal, 1:200), and CD133 (A0219, Abclonal, 1:100), were applied to the sections and left to incubate overnight at 4°C. The slides were then counterstained with light hematoxylin, subjected to dehydration, and covered with slips. The outcomes were evaluated by two pathologists independently, with no access to clinical data, and subsequent analyses encompassed TNM staging and survival assessment. The study was conducted with the written informed consent of the patients and approved by the Institutional Review Committee of East Hospital Affiliated with Tongji University in Shanghai.

### Xenograft assay

The Animal Protection and Use Committee of Tongji University approved all animal experiments. Animal experimentation involved the utilization of 10 BALB/c nude mice, all of the female gender and aged 6 weeks.KYSE-150-FOXC1-LV and KYSE-150-NC-LV were injected subcutaneously into the right abdomen of two groups of mice, purchased from Gempharmatech Co., Ltd. The mice were euthanized, and the tumors were subsequently extracted after 4 weeks for size measurement and weighing. A portion of the tumor tissue was fixed with 10% paraformaldehyde and paraffin-embedded for subsequent immunohistochemical staining analysis, and the rest was used for protein and mRNA extraction.

### Statistical analysis

The in vivo experiments were repeated three times and the final results were taken as the mean ± standard deviation. Statistical comparison analysis was performed by GraphPad Prism 8.0. For survival analysis, the Kaplan-Meier method and log-rank test were employed, and statistical significance was established for *P* values less than 0.05.

### Supplementary information

supplementary figure S1 legends

supplementary figure S1

Original Data File

## Data Availability

The data supporting the findings of this study can be obtained from the corresponding authors, Dr Wu & and Dr Xu, upon reasonable request.
