# Supplementary material for: IGF-1-mediated FOXC1 overexpression induces stem-like properties through upregulating CBX7 and IGF-1R in esophageal squamous cell carcinoma
Source: Cell Death Discov. 2024 Feb 27;10:102. doi: 10.1038/s41420-024-01864-0 (PMC10899262; doi:10.1038/s41420-024-01864-0)
Supplement: Supplementary file 1 — supplementary figure S1 legends [file 41420_2024_1864_MOESM1_ESM.docx]

**Supplementary Figure S1: The expression of CD44 and CD133 is correlated with FOXC1 expression**

1. The expression of CD44 and CD133 correlates with FOXC1 expression.

B. The expression of CD44 is positively correlated with FOXC1 expression.

C. The expression of CD133 positively correlates with the expression of FOXC1.

D. Venn diagram presenting the overlap of upregulated genes in both KYSE-150 and ECA-109 cell lines. (log FC ＜-3).
