# Supplementary figures and images for: IGF-1-mediated FOXC1 overexpression induces stem-like properties through upregulating CBX7 and IGF-1R in esophageal squamous cell carcinoma
Source: Cell Death Discov. 2024 Feb 27;10:102. doi: 10.1038/s41420-024-01864-0 (PMC10899262; doi:10.1038/s41420-024-01864-0)

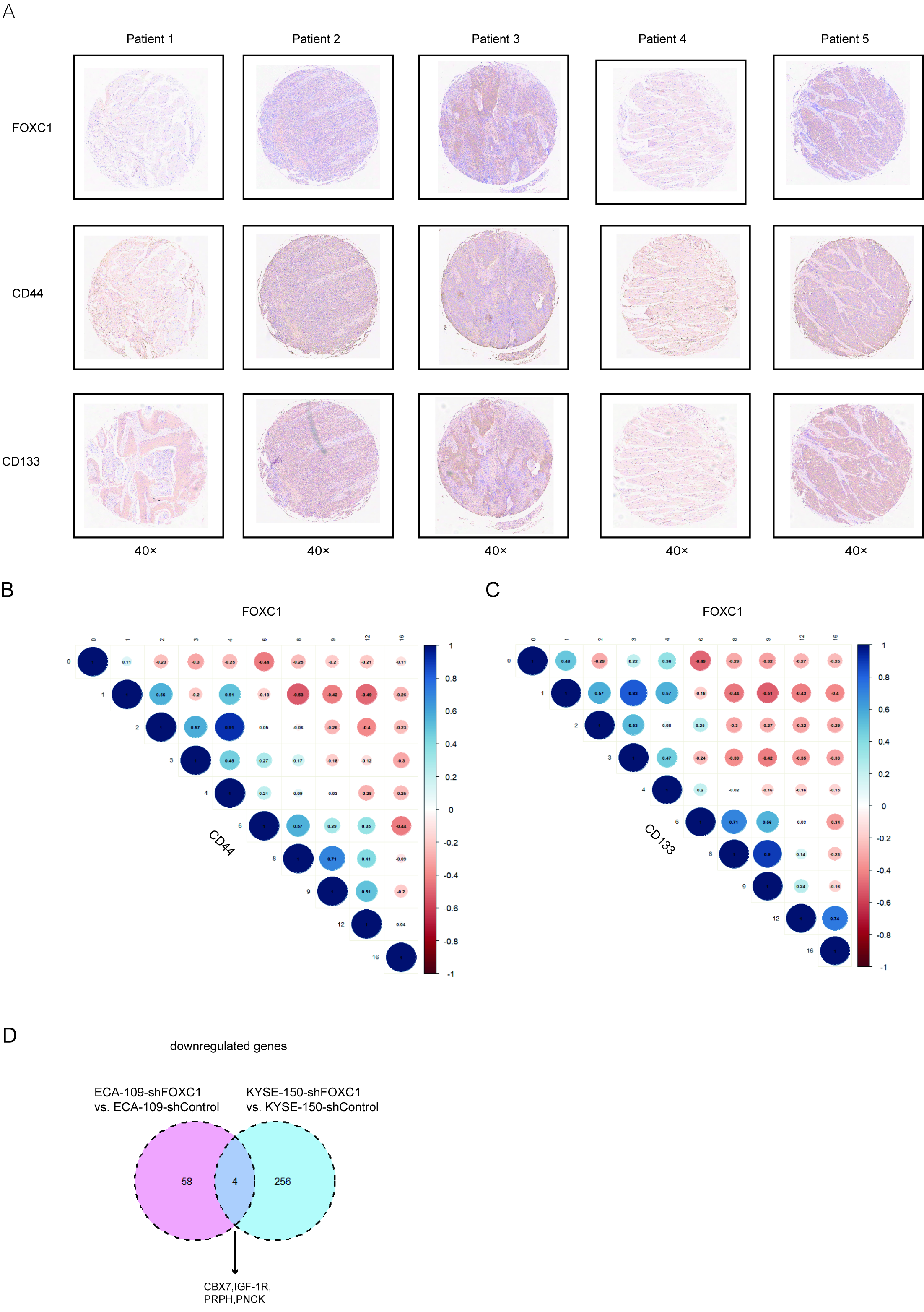

Supplement: Supplementary file 2 — supplementary figure S1 [file 41420_2024_1864_MOESM2_ESM.tif]
