# Supplementary material for: IGF-1-mediated FOXC1 overexpression induces stem-like properties through upregulating CBX7 and IGF-1R in esophageal squamous cell carcinoma
Source: Cell Death Discov. 2024 Feb 27;10:102. doi: 10.1038/s41420-024-01864-0 (PMC10899262; doi:10.1038/s41420-024-01864-0)
Supplement: Supplementary file 3 — Original Data File [file 41420_2024_1864_MOESM3_ESM.pdf]

Figure1 I

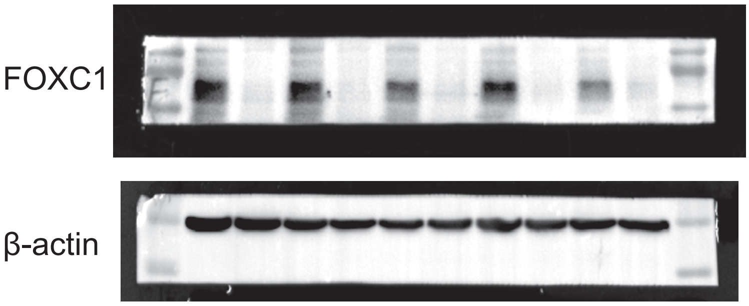

Figure1 J

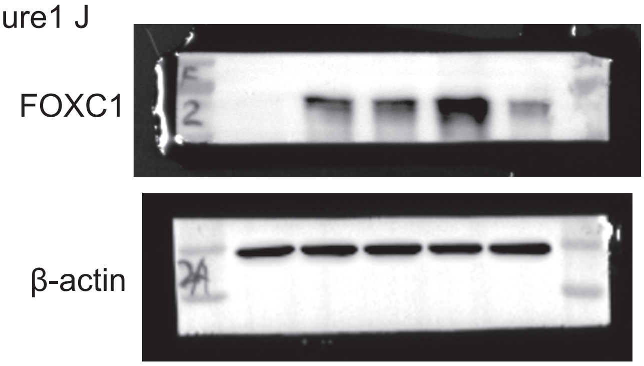

Figure2 A

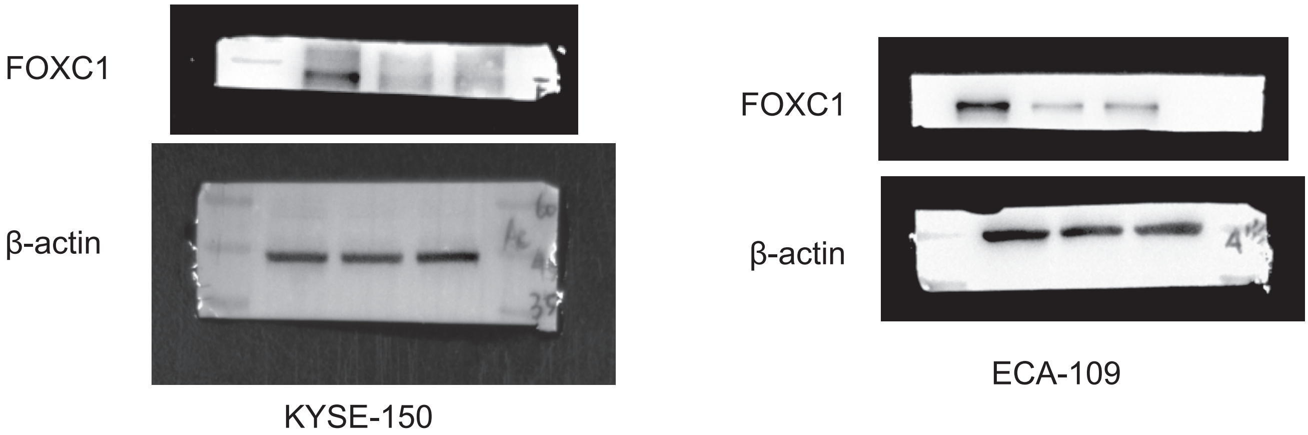

Figure2 H

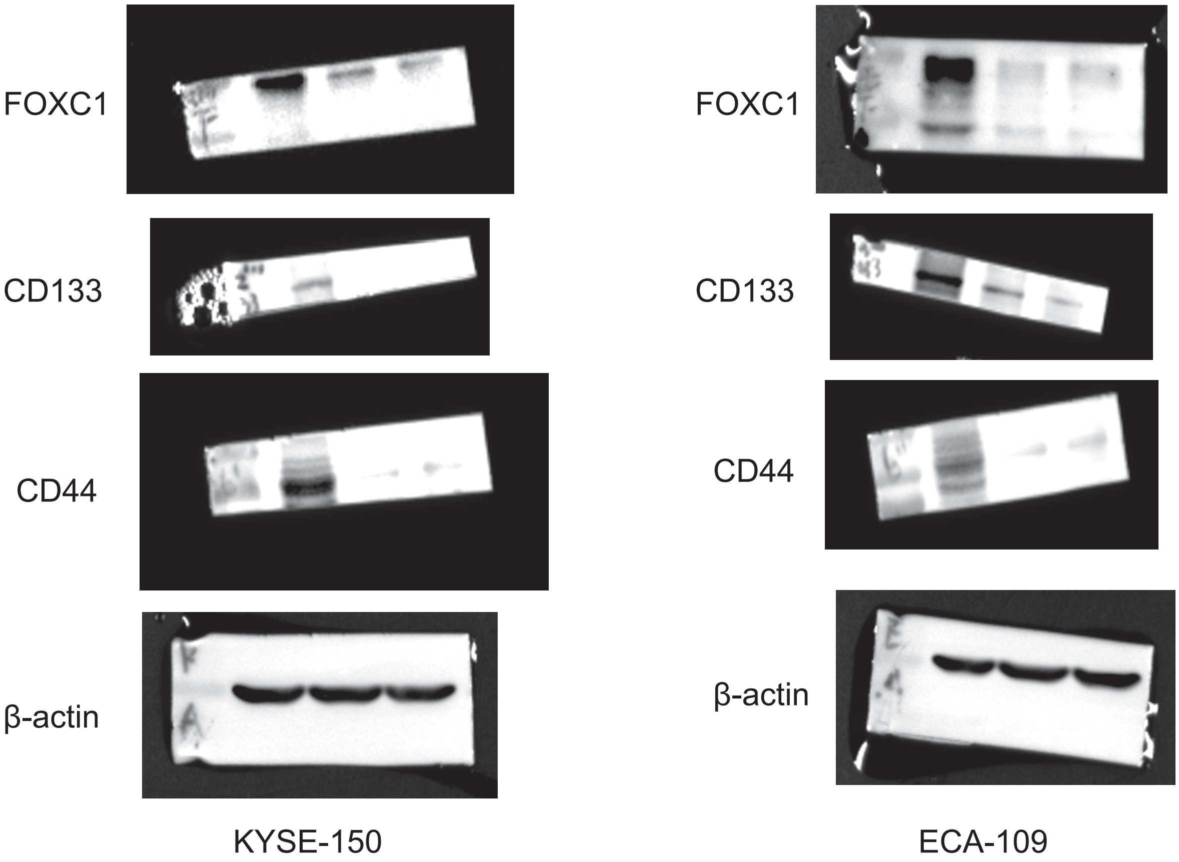

Figure3F

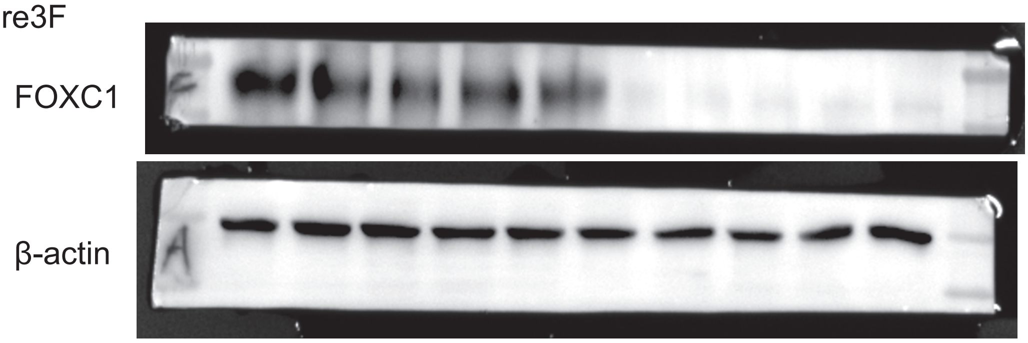

Figure4A

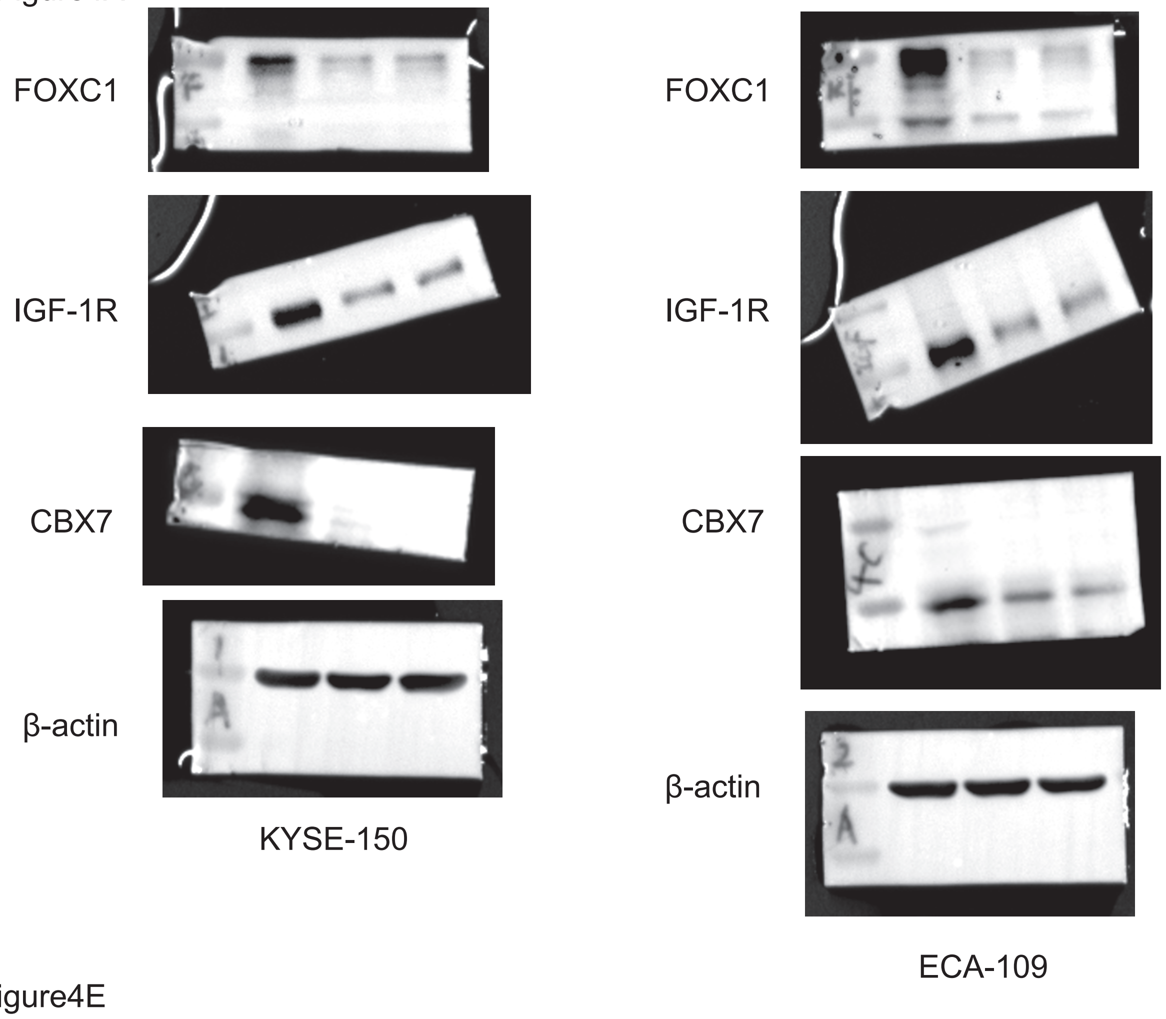

Figure4E

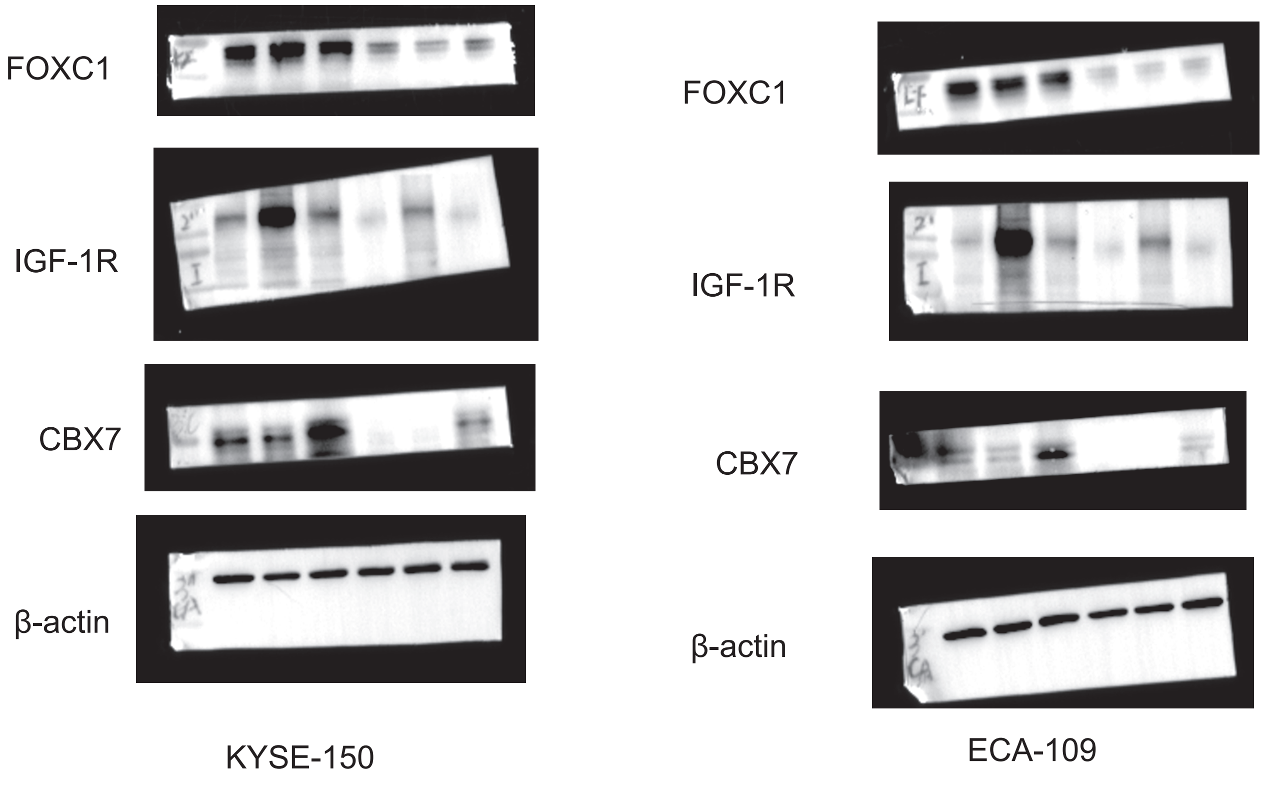

Figure4H

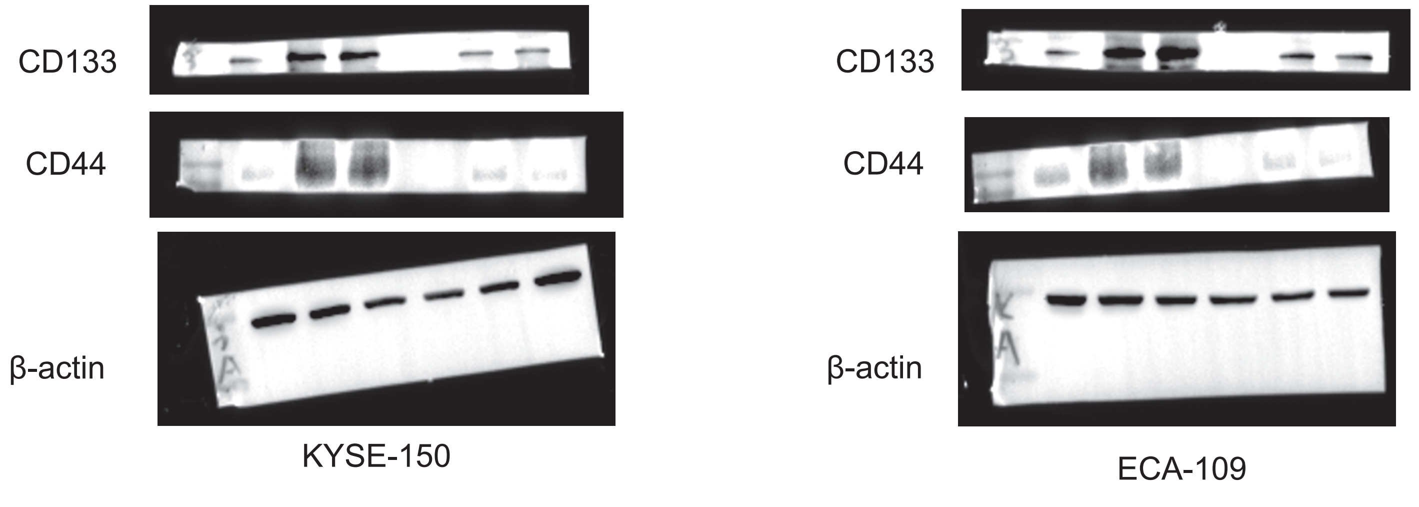

Figure5A:

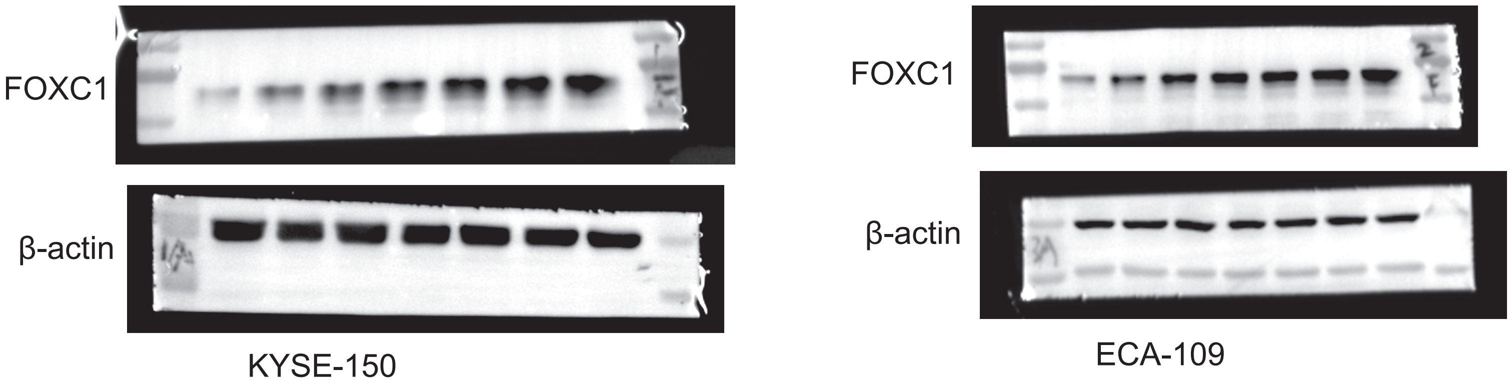

Figure5C:

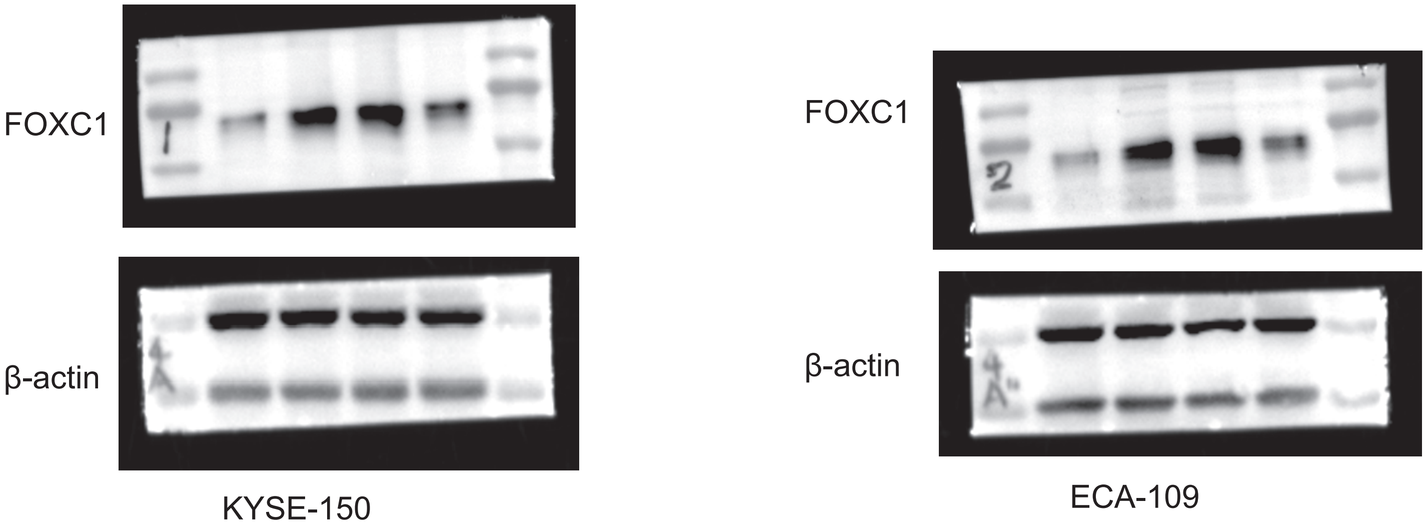

Figure5F:

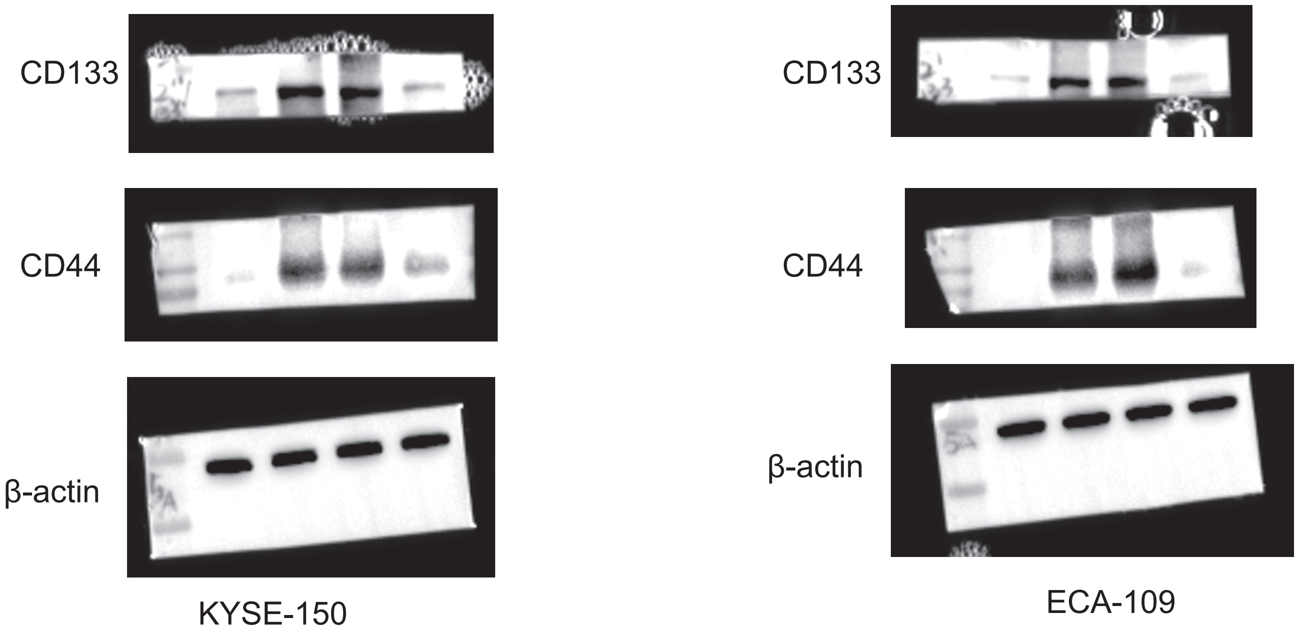

Figure6A:

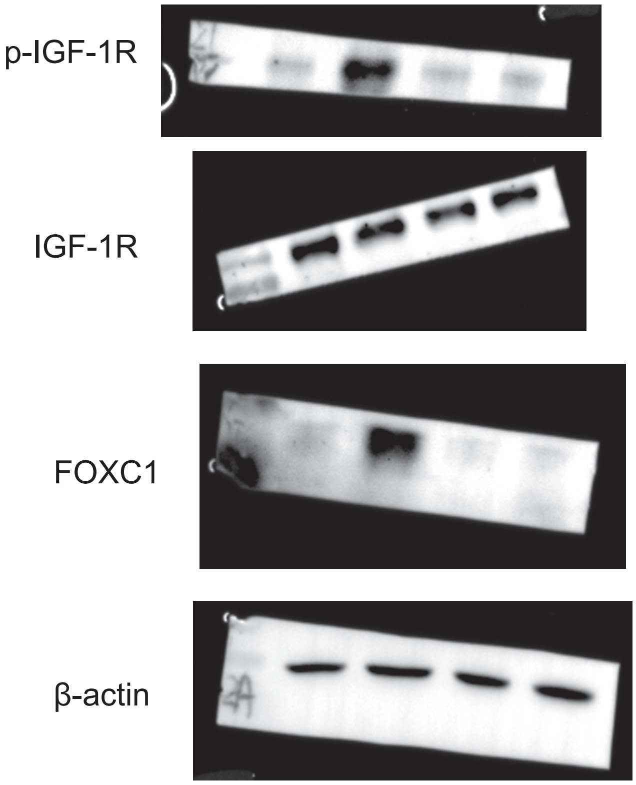

Figure6C:

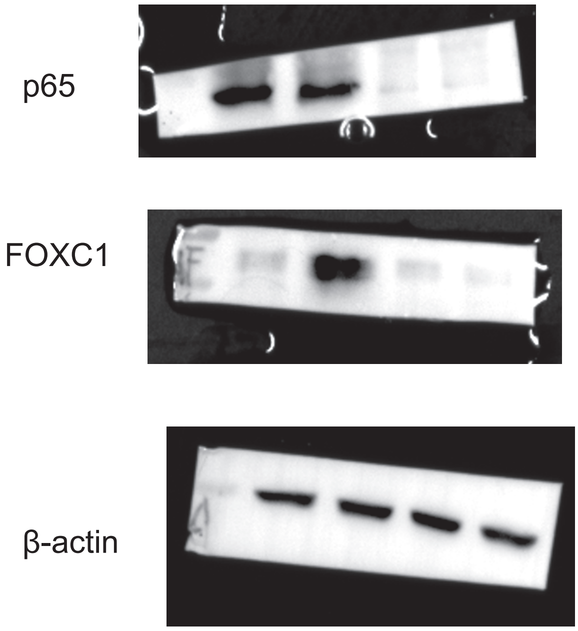

Figure6F:

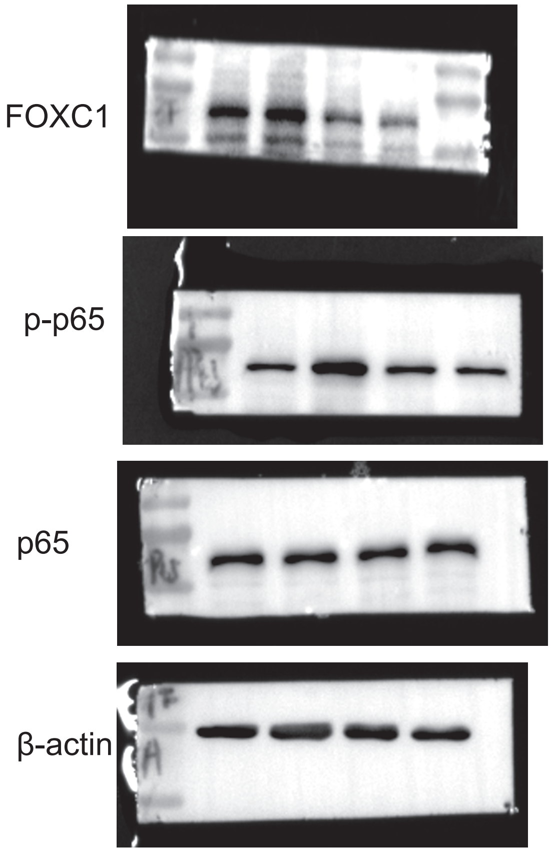

Figure6I:

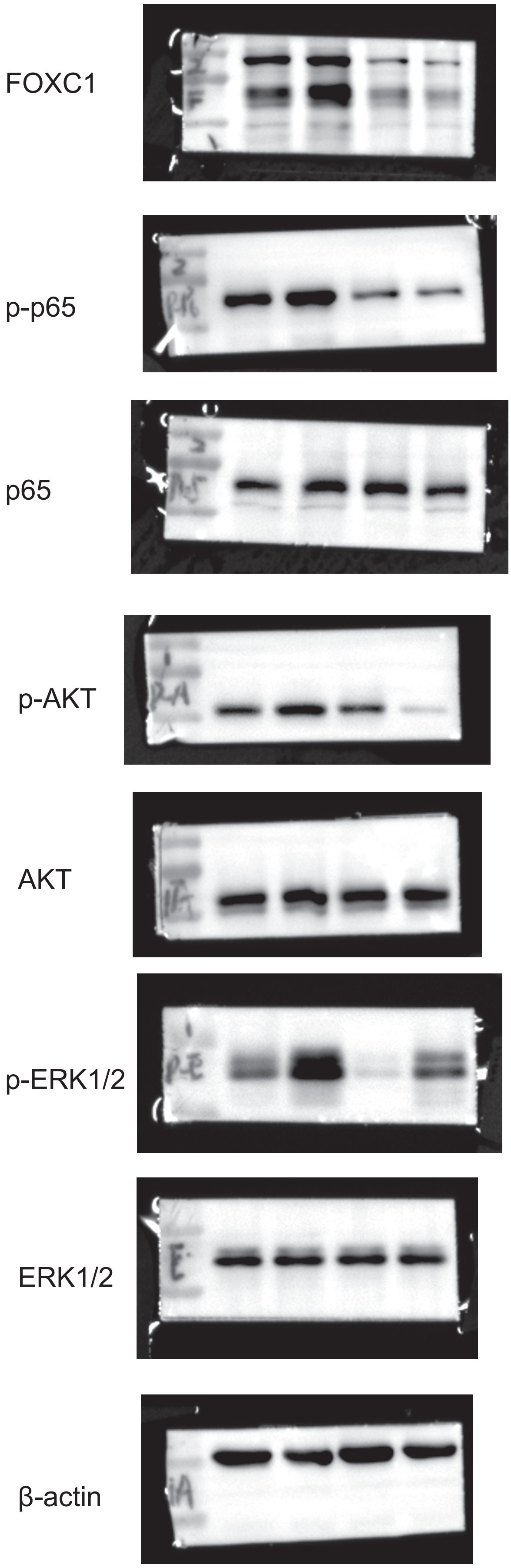

Figure 5C（repeated experiment）:

Auto exposure time

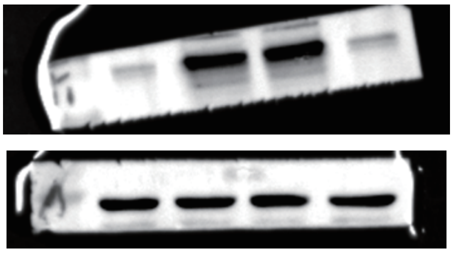

KYSE-150

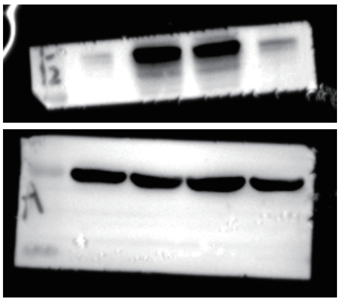

ECA-109

Adjusted exposure time

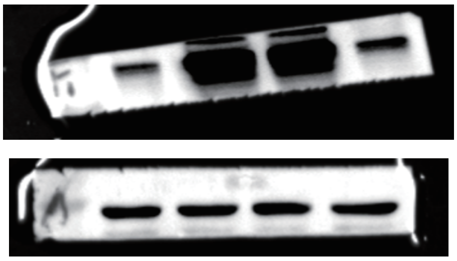

KYSE-150

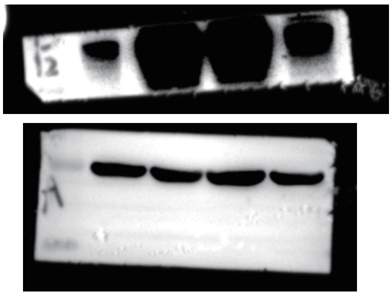

ECA-109

Figure 4H（repeated experiment）:

Auto exposure time

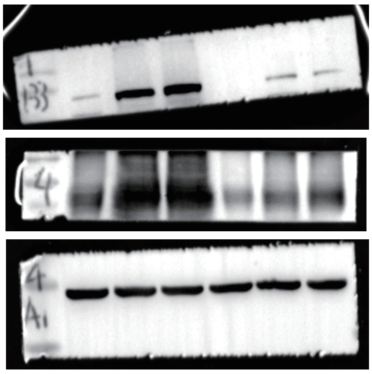

KYSE-150

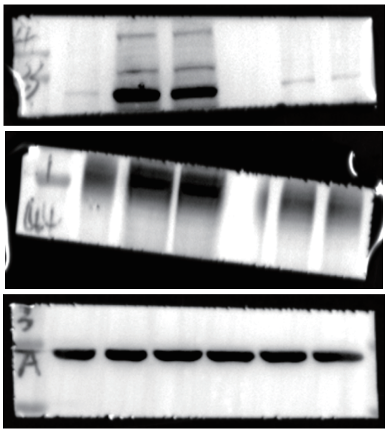

ECA-109

Adjusted exposure time

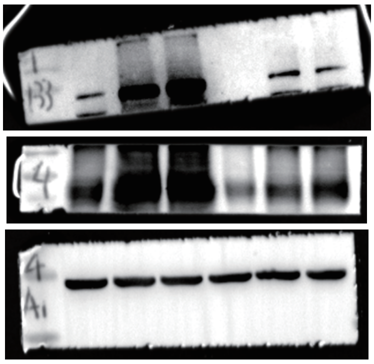

KYSE-150

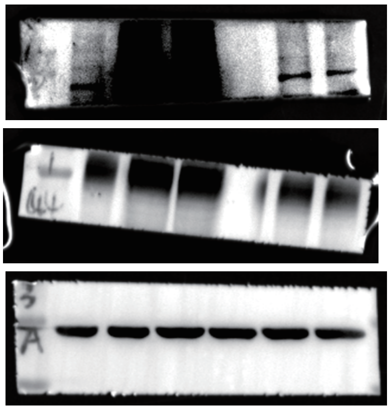

ECA-109
